# Divergent Anabolic Response to Exercise in Young and Older Adult Men-Dependency on Time Frame of Measurement

**DOI:** 10.1093/gerona/glab040

**Published:** 2021-02-04

**Authors:** Søren Reitelseder, Jacob Bülow, Lars Holm

**Affiliations:** 1 Department of Orthopedic Surgery M, Institute of Sports Medicine Copenhagen, Bispebjerg Hospital, Denmark; 2 Department of Biomedical Sciences, Faculty of Health and Medical Sciences, University of Copenhagen, Denmark; 3 School of Sport, Exercise and Rehabilitation Sciences, University of Birmingham, UK

**Keywords:** Alanine, Daily living, Deuterated water, Muscle protein synthesis, Phenylalanine

## Abstract

Older adults’ skeletal muscle has shown to be less responsive to anabolic stimuli as compared to young both in vitro, in short and controlled in vivo settings and in long-term training studies. However, to translate controlled mechanistic findings to long-term adaptations intermediate measures allowing daily life routines with regard to activity and diet would be useful to evaluate physiological interventions. The purpose of this study was to investigate the exercise effect in young and older adults with 2 independent methods to measure muscle protein synthesis rate. Healthy young and old men were recruited to the study protocol where myofibrillar fractional synthesis rate was measured during 2 days allowing normal activities of daily living with D_2_O-labeled alanine and during 4 hours in the overnight fasted state with [^13^C_6_]phenylalanine infusion. During this period 1 leg completed an exercise session every day (exercise leg) while the contralateral leg was kept inactive (normal leg). Both legs were used for activities of daily living. Two-day myofibrillar fractional synthesis rate was significantly higher in the exercise leg in both young and old as compared to normal leg with no age difference. The 4-hour overnight fasted myofibrillar fractional synthesis rate showed that only young exercise leg was significantly higher than normal leg. The present findings support the notion that anabolic resistance exists in the skeletal muscle of healthy older men when evaluated in controlled settings. However, this response is not as clear when measured during daily life where variance is greater, which calls for further investigations in larger cohorts.

The state-of-the-art method to assess muscle protein turnover rates is by tracing stable isotope-labeled amino acids (AAs). Although the very first studies utilized deuterated water (D_2_O) (1), this approach was replaced by infusion of labeled essential AA (EAA), and the *direct incorporation method* for measuring the protein fractional synthesis rate (FSR) became widespread (2). Over the last decades numerous studies have been conducted applying a continuous infusion protocol using D-, ^13^C-, or ^15^N-labeled EAA to determine the muscle FSR over hours (limited to <24 hours). The FSR measurement is dependent on a continuous intravenous tracer infusion and requires rather controlled experimental conditions, which restricts the application of certain physiological interventions. Hence, this approach is valid to assess an acute response to simple and very controlled interventions, but its strengths may be challenged by habitual daily living conditions.

The D_2_O has recently been reintroduced in the field of protein turnover due to its ability to label metabolites in vivo allowing the determination of turnover rates of a variety of substrates (3). AAs are de novo labeled and incorporated into proteins in a manner similar to the *direct incorporation method* (4–6). Due to the enormous pool size of water and the fast equilibration of AA labeling from D_2_O, the precursor enrichment is easily controlled (4), and the D_2_O approach can be applied to measure the gross average tissue FSR across weeks in humans (7). More lately, the use of gas chromatography pyrolysis isotope ratio mass spectrometry (GC-P-IRMS) has allowed the detection of very low abundances of D-labeled AA, which allows muscle FSR to be measured across days (8,9).

While the principles are comparable, the time resolution and experimental conditions for the 2 FSR approaches are distinct and hence can supplement one another and provide insight into different aspects of fluctuations and responses in FSR. This study aimed to compare normal and exercised muscle FSR in young and older healthy men when determined across 2 days during habitual daily life using the D_2_O approach and over 4 hours in the overnight fasted state using the [^13^C_6_]phenylalanine infusion approach.

## Method

### Participants and Baseline Measurements

Eight healthy young and 8 healthy old volunteers were recruited to participate in the study with the following age criteria 18–30 years (young), >65 years (old), body mass index 20–30 kg/m^2^, and not partaking in any habitual sports activity. Study purpose, design, and possible risks were explained to each participant before informed written consent to participate was given. The study protocol adhered to the Declaration of Helsinki II and was approved by the local Ethics Committee of the Capital Region of Denmark (H-1-2012-102). Of the recruited participants, 8 young and 7 old participants completed all study protocol procedures and were included in the analyses. Participants had their anthropometrics determined including body composition with dual-energy X-ray absorptiometry (DPX-IQ software version 4.6c; Lunar, Madison, WI). One repetition maximum (1 RM) for leg press and leg extension exercises was also determined. Participant characteristics are displayed in Table 1.

**Table 1.** Participant Characteristics

	Young (*n* = 8)	Old (*n* = 7)
Age (y)	23 ± 3	70 ± 4
Height (m)	1.79 ± 0.05	1.78 ± 0.06
Weight (kg)	77.5 ± 7.5	83.6 ± 5.9
Body mass index (kg/m^2^)	24.1 ± 2.5	26.5 ± 1.9
Lean body mass (kg)	59.4 ± 5.6	60.6 ± 3.8

*Note*: Participant characteristics (means ± *SD*), except for age, no significant differences were found between the groups.

### Experimental Protocol

**Figure 1.**
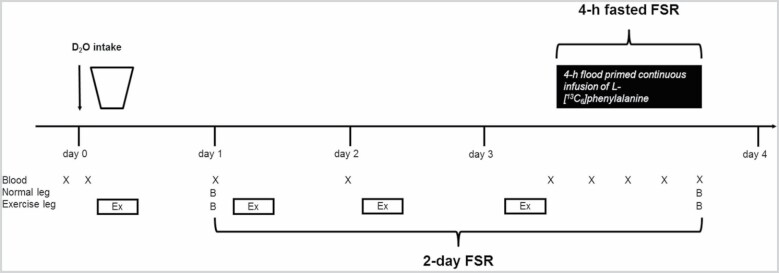
Experimental protocol. The experimental protocol included the D_2_O intake on day 0 followed by 1-legged exercise sessions on day 0, 1, 2, and 3. Venous blood samples were collected on day 0 before and 2 hours after the D_2_O intake and on day 1, 2 (not fasted) and on day 3 in the overnight fasted state before the 4-hour flood primed continuous infusion of L-[^13^C_6_]phenylalanine and once every hours for 4 hours. Muscle biopsies were collected in both the normal and exercised legs on day 1 before the exercise session and on day 3 after the exercise session and following 4 hours of tracer infusion. FSR = fractional synthesis rate.

All participants underwent the experimental protocol as illustrated in Figure 1. D_2_O was provided on day 0 (10 am) with a background blood sample taken just before. The amount of D_2_O was adjusted to 0.65% body water, which was based on measurements of lean body mass. Ninety-nine percent D_2_O (Cambridge Isotope Laboratories, Tewksbury, MA) was mixed with normal tap water to ~70%. A second blood sample was taken 2 hours after the D_2_O intake. Thereafter, the first heavy resistance exercise session was performed, which consisted of unilateral leg press and leg extension exercises. Both exercises were performed as 4 sets of 8 repetitions at 70% of 1 RM with 3 minutes of rest between the sets. The exercise leg was randomly assigned but balanced in groups with young and older men between dominant and nondominant legs.

On day 1 (10 am) a blood sample was taken and muscle biopsies (in vastus lateralis) were taken in both legs. Thereafter, the second exercise session was performed. On day 2 (10 am) a blood sample was taken and the third exercise session performed. On day 3 (10 am) participants arrived to the laboratory after an overnight fast and performed the fourth and final exercise session. Thereafter, antecubital venflons were inserted in both arms and a flood primed continuous infusion of L-[ring-^13^C_6_]phenylalanine and unlabeled phenylalanine (Cambridge Isotope Laboratories) was started (10), and blood samples were taken at 1, 2, 3 and 4 hours of infusion. Muscle biopsies were obtained from both legs after 4 hours of infusion. All blood and muscle biopsies were treated and stored as previously described (10). This experimental design provided 2-day habitual and 4-hour fasted myofibrillar FSR.

### Tracer Analyses

Venous plasma and muscle biopsies were prepared and analyzed as previously described (10–12). Plasma alanine and phenylalanine enrichments were measured by gas chromatography tandem mass spectrometry (GC-MS/MS, Thermo Scientific, TSQ Quantum, San Jose, CA). Myofibrillar protein [^13^C_6_]phenylalanine abundance was analyzed by GC combustion isotope ratio mass spectrometry (GC-C-IRMS, Finnigan Delta Plus, Bremen, Germany) and [D]alanine abundance was analyzed GC-P-IRMS (GC Combustion III, Delta Plus XL; Thermo Finnigan, Bremen, Germany). Details of these methods have been described previously (10–12). FSR was calculated as FSR (%/h) = [(Δ*E*_protein_)/(*E*_precursor_ × Δ*t*)] × 100%, where Δ*E*_protein_ is the change in protein-bound labeled alanine or phenylalanine, *E*_precursor_ is the mean alanine or phenylalanine enrichment in the incorporation periods defined by Δ*t*.

### Statistical Analyses

Participant characteristics were compared by unpaired *t* tests. Plasma alanine and phenylalanine enrichments and the myofibrillar FSR were compared by 2-factor ANOVA with time and exercise as repeated and group as nonrepeated measures. In case of main significant effects, Student–Newman–Keuls post hoc tests were performed. All values are means ± *SE* except participant characteristics, which are means ± *SD*. Effects with *p* < .05 were considered statistically significant. Statistical analyses were performed in GraphPad Prism 7.00 (GraphPad Software, Inc., La Jolla, CA).

## Results

**Figure 2.**
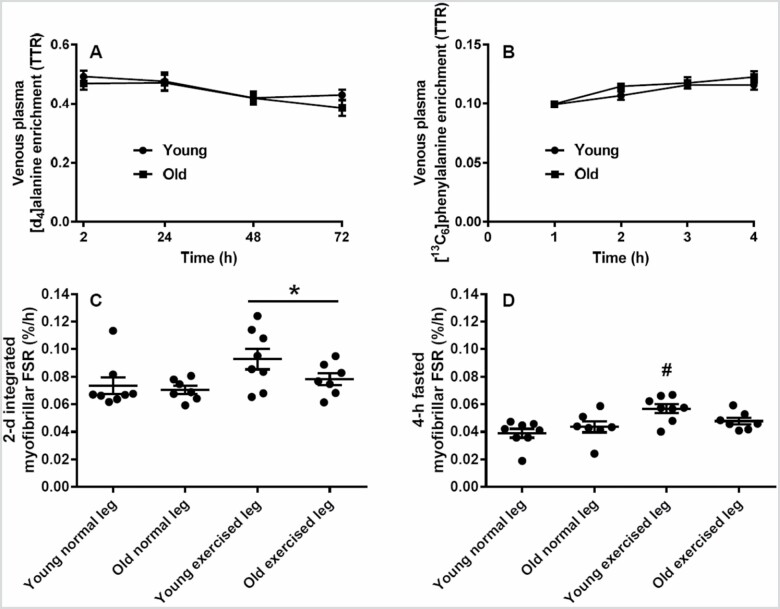
Precursor enrichments and myofibrillar synthesis rates. Mean (± *SE*) venous plasma alanine (A) and phenylalanine (B) enrichment and myofibrillar FSR based on [D]alanine (C) and [^13^C_6_]phenylalanine (D). The plasma alanine and phenylalanine enrichments show a significant effect of time overall (*p* < .001), the 2-day myofibrillar FSR show a significant effect of state (*p* < .01; * exercised leg higher than normal leg [*p* < .05]), and the 4-hour fasted myofibrillar FSR show a significant interaction (*p* < .05; ^#^ young exercised leg higher than young normal leg [*p* < .05]); FSR = fractional synthesis rate; TTR = tracer-to-tracee ratio.

The D_2_O-labeled [D_4_]alanine (Figure 2A) and [^13^C_6_]phenylalanine (Figure 2B) precursor enrichment over the 2 different tracer incorporation periods showed both a general effect of time (*p* < .001), but with no difference between young and old.

The 2-day myofibrillar FSR was significantly higher in the exercise leg in both young (2.23 ± 0.18%/d [mean ± *SE*], 0.093 ± 0.008%/h) and old (1.88 ± 0.11%/d, 0.078 ± 0.004%/h) as compared to normal leg (young: 1.76 ± 0.15%/d, 0.073 ± 0.006%/h; old: 1.69 ± 0.07, 0.070 ± 0.003%/h, *p* < .01) with no age effect. The 4-hour overnight fasted myofibrillar FSR showed that only in young males the exercised leg (0.057 ± 0.003%/h) was significantly higher than young normal leg (0.039 ± 0.003, *p* < .05), whereas old exercise leg (0.048 ± 0.002) and old normal leg (0.044 ± 0.004%/h) showed no difference. No significant differences were observed between young and old normal leg myofibrillar FSR irrespective of the measurement period and method. The 2-day myofibrillar FSR (Figure 2C) and the 4-hour overnight fasted myofibrillar FSR (Figure 2D) are shown as individual values with mean and *SE* in %/h.

## Discussion

The main findings in this study were that we verified a similar nonexercised myofibrillar FSR between ages independent of time frames of measurement (2-day daily living vs 4-hour fasting). However, older men only increased muscle FSR when measured across days in daily living conditions while both time frames detected an increase in the young men. Therefore, the nature of the different and independent methods to estimate muscle protein synthesis warrants divergent interpretation of the results.

### Daily Life Muscle Protein Synthesis

Normal daily life rates of myofibrillar FSR were equal in young and older adult men. This observation was irrespective of whether the measurement was performed over 2 days in a fully nourished muscle with normal activity of daily living (ADL), but with no strenuous physical requirements or performed over 4 hours in the overnight fasted state. It has previously been shown that the overnight fasting and resting muscle protein synthesis and turnover rate is not markedly changed with age (13,14), although also contrasting data are reported (15). However, despite being equal between young and old men, the normal rates are different when measured over 2 days during daily life with included factors as ADL, nutrition, and sleep than during 4 hours in the overnight fasted and rested state. The 2-day FSR should be considered the “normal” condition, whereas the 4-hour overnight fasted and rested state should be considered the “basal” state. As expected, the FSR is lowest in the basal as compared to the normal state, suggesting that a balanced energy intake according to official recommendations as well as moderate ADL (walking, cycling, climbing stairs as well as sitting and laying) even when combined with periods of sleep elevates muscle protein synthesis rates above the overnight fasted, resting condition (16). The obvious explanation is that a 2-day period includes several intervals of elevated FSR rates due to habitual intake of meals and physical ADL, which add up to greater gross mean rates. One previous study (17) has compared the D_2_O-labeled alanine and [^13^C_6_]phenylalanine tracer approaches. The study used the D_2_O-labeled alanine method over few hours and hence, found values exactly comparable to the EAA approach, emphasizing that the tracer approaches are comparable and that the present divergence is ascribed to impact of normal daily living versus fasting, resting condition. As showed with this study, the advantage given with the D_2_O-labeled alanine method is that it allows measurements of protein FSR over prolonged periods of time with normal daily living activities, such as meal intake, ADL, and sleep.

### Muscle Protein Synthesis Response to Exercise

The muscle adapts with growth to prolonged resistance training in both young (18,19) and old age (20,21), and the mechanism at least in muscles of young individuals is a stimulation of the muscle protein synthesis in a period after completion of muscle contractions (22) and Figure 2D. In contrast, we could not detect an acute enhancement in myofibrillar FSR in the older muscle. Such an abolished or impaired responsiveness to acute exercise in aging muscle has been shown previously (13,23,24). The existence of a diminished response is strengthened with our results by the fact that we preconditioned the muscle prior to determining the muscle FSR with 3 resistance exercise bouts performed daily prior to trial on experimental day 3. Hence, not even repeated sessions of heavy load exercise sessions exhibit an increased muscle protein synthesis in older men.

Extending the muscle FSR measurement period is not common. In young people, 24-hour whole-body protein turnover rate was not affected by completion of 1-hour moderate-intensity cycling exercise (25). However, enhanced basal synthesis up to 48-hour postresistance exercise suggests a prolonged stimulatory effect in young muscle (22). Our results on the heavy resistance exercise myofibrillar FSR response during 2 days showed effect in both young and old men, but the exercise response variance appeared to be larger as compared to the measurements in the controlled 4-hour fasted setup.

## Conclusion

The present findings support the notion that anabolic resistance exists in the skeletal muscle of healthy older men when evaluated over a short time frame in a controlled setting. However, this response is not as clear when measured in a daily life setting and deserves further investigations possibly also to include protein breakdown rates to reveal clinical significance. The advantage of the D_2_O-labeled alanine method to assess muscle protein synthesis during periods of daily normal living activities can aid future studies to translate molecular mechanistic signaling findings to long-term adaptations in muscle mass and physical function.
